# Ascorbate-dependent and ascorbate-independent Mn porphyrin cytotoxicity: anticancer activity of Mn porphyrin-based SOD mimics through ascorbate-dependent and -independent routes

**DOI:** 10.1080/13510002.2021.1917214

**Published:** 2021-04-26

**Authors:** Bader Hasan, Artak Tovmasyan, Ines Batinic-Haberle, Ludmil Benov

**Affiliations:** a Department of Biochemistry, Faculty of Medicine, Kuwait University, Kuwait City, Kuwait; b Department of Neurobiology, Ivy Brain Tumor Center, Barrow Neurological Institute, Phoenix, AZ, USA; c Department of Radiation Oncology, Duke University School of Medicine, Durham, NC, USA

**Keywords:** Anticancer activity, tumoricidal, hydrogen peroxide, cellular uptake, metalloporphyrin, reactive oxygen species, membrane damage, ascorbate

## Abstract

**Objective:**

The aim of this study was to investigate how modifications at the periphery of the porphyrin ring affect the anticancer activity of Mn porphyrins (MnPs)-based SOD mimics.

**Methods:**

Six compounds: MnTE-2-PyP with a short ethyl chain on the pyridyl ring; MnTnHexOE-2-PyP and MnTnOct-2-PyP with linear 8-atom alkyl chains, but the former with an oxygen atom within the alkyl chain; MnTE-2-PyPhP and MnTPhE-2-PyP with pyridyl and phenyl substituents, were investigated. Cytotoxicity was studied using pII and MDA-MB-231 cancer cell lines. Viability was assessed by the MTT (3-[4,5-dimethylthiazol-2-yl)]-2,5-diphenyltetrazolium bromide) assay and cell proliferation was determined by the sulforhodamine B assay.

**Results:**

Cellular uptake was increased with the increase of the lipophilicity of the compounds, whereas reduction potential (*E*_½_) of the Mn(III)/Mn(II) redox couple shifted away from the optimal value for efficient redox cycling with ascorbate, necessary for ROS production. Amphiphilic MnPs, however, exerted anticancer activity by a mechanism not involving ROS.

**Conclusion:**

Two different processes account for MnPs cytotoxicity. MnPs with appropriate *E*_½_ act via a ROS-dependent mechanism. Amphiphilic MnPs with suitable structure damage sensitive cellular constituents, leading to the suppression of proliferation and loss of viability. Design of compounds interacting directly with sensitive cellular targets is highly promising in the development of anticancer drugs with high selectivity and specificity.

## GRAPHICAL ABSTRACT

**Figure:**
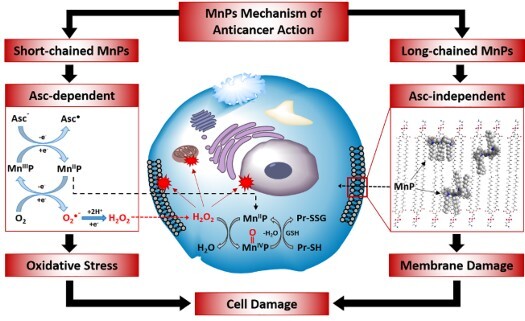

## Introduction

Mn-porphyrins (MnPs) have been developed as SOD mimics and redox-active antioxidants [1–3]. In the presence of natural reductants like ascorbate, however, MnPs exert cytotoxicity [4–7]. The cytotoxic effect has been attributed to redox-cycling, leading to the production of reactive oxygen species (ROS), which damage and eventually kill the cells [4,5,8,9]. Based on the investigations of Mn(III) *meso*-tetrakis(*N*-ethylpyridinium-2-yl)porphyrin (MnTE-2-PyP), Mn(III) *meso*-tetrakis (*N*-n-hexylpyridinium-2-yl)porphyrin (MnTnHex-2-PyP) and their *meta* analogs on cancer cell lines, it has been concluded that lipophilicity, size and shape of the molecule do not play a substantial role in MnP toxicity when the reducing agent (ascorbate) was added exogenously [4]. Under such conditions, cellular uptake of MnPs has a minor impact, because toxicity was predominantly due to hydrogen peroxide produced outside the cell. Since the generation of H_2_O_2_ depends on the reducibility and reoxidation of the compound, the redox potential of MnP, which determines the redox-cycling and ROS production, is considered the main determinant of cytotoxic efficacy [4].

Ascorbate, at high (∼15 mM) concentrations, resulting in the production of cytotoxic ascorbate radical and hydrogen peroxide, has been used for anticancer treatment (for review, see [10]). Achievement of such therapeutic ascorbate concentrations, however, requires intravenous delivery because oral doses provide only up to 100–200 µM. Previous research has demonstrated that in the presence of MnPs, similar anticancer effect can be achieved at lower ascorbate concentrations [4–6,11–13].

Preferential uptake and accumulation of porphyrins is a common feature of cancerous tissues and has been used for tumor localization [14] and photodynamic anticancer treatment [15–18]. Selective uptake of MnPs by cancer cells, leading to intracellular redox-cycling and ROS production, could provide a way for highly specific, light-independent cancer treatment. Our previous investigations revealed the key properties of a metalloporphyrin molecule, which determine its cellular uptake and subcellular distribution [19–21]. If a modification of a MnP molecule combines the favorable redox potential with high uptake and accumulation in cancer cells, a selective and specific cancer treatment could be achieved.

While redox-cycling of MnPs giving rise to H_2_O_2_ has been studied in detail, the impact of molecular properties, such as lipophilicity, size, shape, and flexibility of the molecule, has not been extensively explored [8, 21–26].

The aim of this study was to investigate how modifications at the periphery of the porphyrin ring, which affect lipophilicity, charge distribution, thermodynamic property and three-dimensional shape of the molecule influence its cytotoxicity against cancer cell cultures.

## Materials and methods

### Cell cultures

The following cancer cell lines were used in this study: pII (obtained from Prof. Yunus Luqmani, Faculty of Pharmacy, Kuwait University) and MDA-MB-231 (ATCC® HTB­26™). pII is an estrogen receptor (ER)-negative breast cancer cell line derived from MCF-7 does not respond to estradiol or tamoxifen [27–29]. MDA-MB-231 is an ER, progesterone receptor (PR) and HER2 (human epidermal growth factor receptor 2) negative and metastatic epithelial breast cancer cell line [30].

Monolayer cell cultures were grown in RPMI 1640 (Gibco) supplemented with 10% fetal bovine serum (FBS), 2 mM l-glutamine (Gibco) and 100 U mL^−1^ penicillin/streptomycin as antibacterial agents in a humidified incubator at 37°C and 5% CO_2_ and 95% air. Cells were passaged at 80–90% confluence, the growth medium was discarded, and cells were washed with PBS. Cells were then detached by the addition of trypsin-EDTA (Gibco) for 1–2 min. Fresh medium, amounting to 10× the trypsin volume, was added to stop the digestive action of trypsin. Cells were then either subcultured or used for experiments. Cell counting was performed with an improved Neubauer hemocytometer and trypan blue was used to differentiate between viable and non-viable cells. Negative control for MnP were cells only and negative controls for the effect of MnP and ascorbate redox cycling was ascorbate alone. For cell membrane integrity experiments, positive controls of heat killed cells and cells treated with 1 mM hydrogen peroxide were used.

### Manganese porphyrins

**Figure 1.**
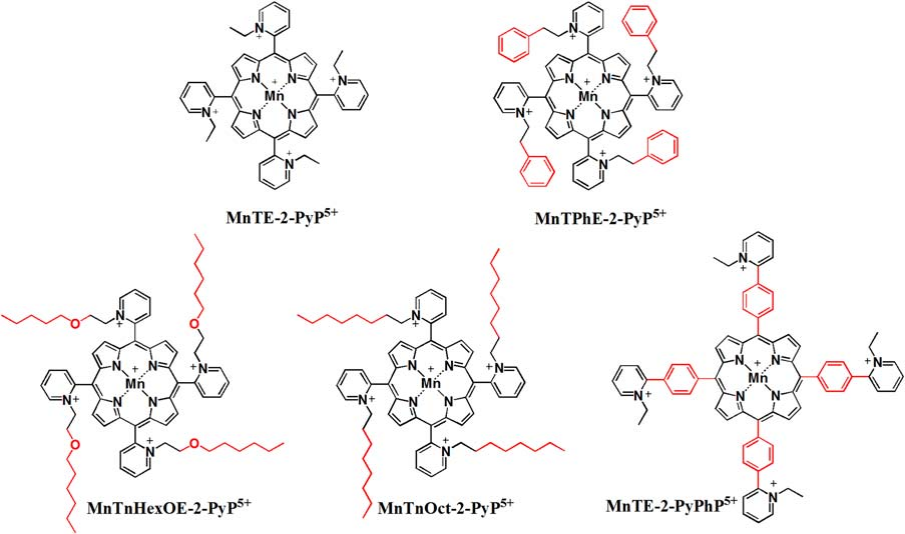
The structures of MnPs used in this study. Note: The charges in the MnP abbreviations are omitted throughout the text for clarity.

Six compounds with different *meso* substituents were used, and their structures are shown in Figure 1. MnTE-2-PyP has a short ethyl chain on the *ortho* position of the pyridyl ring. MnTnHexOE-2-PyP and MnTnOct-2-PyP both have linear 8 carbon alkyl chains in the *ortho* position of the pyridyl ring, but the former has an oxygen atom within the alkyl chain. MnTE-2-PyPhP and MnTPhE-2-PyP have a pyridyl and phenyl substituents, but differently organized. The former has a phenyl ring preceding the pyridyl ring, while the latter bears phenylethyl moiety at pyridyl ring. All of these different substituents result in different properties that are summarized in Table 1 [6]. The synthesis, purification, and characterization by means of thin-layer chromatography, elemental analysis, ESI-MS, and UV-Vis spectroscopy of the compounds used in this study (Figure 1) have been performed according to the previously published methods described in detail elsewhere [31].

**Table 1.** Properties of MnPs. The lipophilicities (log *P_ow_*), reduction potentials for Mn(III)/Mn(II) redox couple (*E*_1/2_ vs. NHE), relative molecular masses, rate constants for H_2_O_2_ dismutation (*k*_cat_ (H_2_O_2_)), and initial rates of ascorbate oxidation to ascorbyl radical of the MnPs used in the study [31].

Manganese porphyrin	Lipophilicity, log *P_ow_*	Reduction potential, *E*_1/2_ vs. NHE (mV)	Relative molecular mass	Ascorbate oxidation rate, *v*_o_ (nM s^−1^)	*k*_cat_ (H_2_O_2_), (M^−1^ s^−1)^
MnTE-2-PyP	−7.67	228	965.1	312.84	63.32
MnTPhE-2-PyP	−5.9	259	1269.5	147.21	23.54
MnTE-2-PyPhP	−5.51	−65	1269.5	18.24	21.1
MnTnOct-2-PyP	−2.27	340	1301.8	54.29	27.62
MnTnHexOE-2-PyP	−1.67	313	1365.8	76.33	34.66

### Cellular uptake of MnPs

Uptake of MnPs by cancer cells was determined as previously described with slight modifications [32]. In brief, 3 × 10^5^ cells per well were seeded into a 6-well plate and allowed to attach overnight. MnPs (5 µM) were added and the cells were incubated for 24 h. At the end of the incubation period, cells were washed with PBS and solubilized overnight at 37°C by treatment with 2% SDS dissolved in distilled water. Cellular accumulation of MnP was determined by recording absorption spectra and determining the area under the peak at the Soret band. MnP concentration was calculated using standard curves. For each compound, a standard curve was built by dissolving each MnP in 2% SDS.

Intracellular MnP accumulation was normalized to protein concentration. Cellular protein was assayed by the modified Lowry method [33].

## Cytotoxicity assays

### MTT test

Cell viability was assessed by the MTT (3-[4,5-dimethylthiazol-2-yl)]-2,5-diphenyltetrazolium bromide) assay. In brief, 1.5 × 10^4^ cells were loaded into sterile 96-well microplates containing 100 µL of RPMI-1640 medium and incubated for 24 h for cells to adhere. MnPs were added at 1 and 5 µM final concentrations. Where indicated, freshly prepared sodium ascorbate was added to a final concentration of 1 mM immediately after the addition of MnP.

Since ascorbate can directly reduce MTT to formazan, cells were thoroughly washed, and fresh medium was added to each well. MTT reagent (5 mg mL^−1^ in PBS) was added to a final concentration of 10% and plates were incubated in the dark for 3 h in a humidified 37°C CO_2_ incubator. Formazan crystals were solubilized for 24 h at 37°C with 100 µL of 10% SDS dissolved in 0.01 M HCL. The absorbance of each well was measured at 560 nm (formazan) and 650 nm (background) [34] using a microplate reader (CLARIOstar, BMG LABTECH Inc., USA).

### Sulforhodamine B assay

The effect of MnP and ascorbate on cell proliferation was investigated using the sulforhodamine B (SRB) assay [35]. In brief, 1.5 × 10^4^ pII cells were loaded into sterile 96-well microplates containing 100 µL of complete RPMI-1640 medium and incubated for 24 h to adhere. MnPs were added at 1 and 5 µM final concentrations and, where indicated, 1 mM of freshly prepared sodium ascorbate was added. Twenty-four and 48 h after treatment the cells were fixed with ice cold trichloroacetic acid (10% final concentration) and incubated at 4°C for 1 h. The microplates were then washed 5 times with deionized water and stained with SRB dye (0.4% dissolved in 1% acetic acid) for 25 min at room temperature. Wells were then washed 5 times with 1% acetic acid to remove any unbound dye and air dried at room temperature. Bound dye was solubilized by the addition of 100 µL of 10 mM Tris base and gently mixed to obtain a homogenous solution. Absorbance was read at 510 nm (dye) and 690 nm (background) using a microplate reader.

### Assessment of membrane integrity

The integrity of the plasma membrane was assessed by exposure of cells to the membrane impermeant dye, propidium iodide (PI); positive staining indicates damaged membrane. PII cells (0.7 × 10^−6^) were seeded into sterile 25 cm^3^ flask containing RPMI 1640 medium and were incubated 24 h to adhere. MnP alone or MnP plus ascorbate was added and incubated for 24 h. H_2_O_2_-treated and heat-treated cells were used as positive controls. At the end of the incubation, floating cells were collected, and the adherent cells were trypsinized. After centrifugation, the cell pellet was resuspended in 500 µL of PBS. Propidium iodide was then added at a concentration of 1 µg mL^−1^ to 100 µL of cells, cell suspension was diluted to 500 µL with PBS and incubated in the dark for 20 min. Samples were then analyzed using a Beckman Coulter CYTOMICS FC 500 series cytometer.

All experiments were repeated at least two times, each sample in triplicate. Results are presented as mean ± S.D.

## Results

Previous studies have shown that killing of cancer cells by ascorbate is dependent on the production of H_2_O_2_ in the presence of transition metal, such as Mn and Fe (or their complexes), in the growth medium [8,36–38]. In order to compare the susceptibility of different cell lines to ascorbate toxicity, two different breast cancer cell lines, pII and MDA-MB-231 grown in the same medium, were tested.

**Figure 2.**
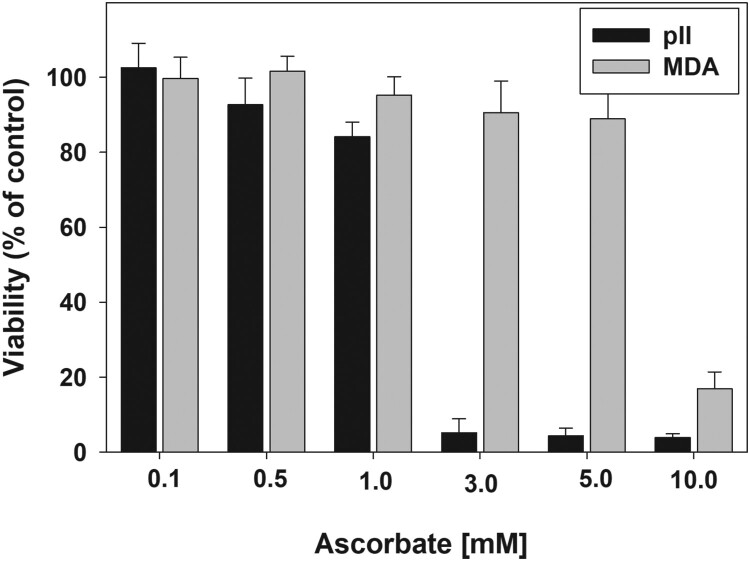
Sensitivity of pII and MDA-MB-231 cell lines to ascorbate. Note: Cells were treated with 0.1–10 mM ascorbate for 24 h. After the treatment, the medium was removed, cells were washed gently with PBS and 100 μL of fresh medium was added to each well. Viability was determined by the MTT assay. Data are presented as mean ± S.D.

Figure 2 shows that the sensitivity of the cancer cell lines toward toxicity of ascorbate varies dramatically. The MTT reduction to formazan by pII cells was almost completely suppressed by 3 mM ascorbate. Yet for MDA-MB-231 cell line such an effect was not achieved even at 10 mM ascorbate. These results indicate that in addition to the growth medium, the toxicity of ascorbate strongly depends on the nature of the cancer cell line.

**Figure 3.**
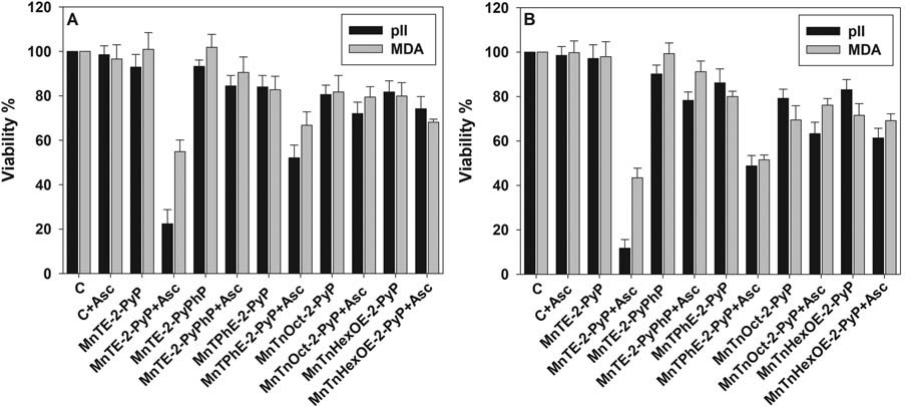
Sensitivity of pII and MDA-MB-231 cell lines to MnP/ascorbate cytotoxicity. Note: Cytotoxicity of MnP/ascorbate redox cycling on pII and MDA-MB-231 cells. Cells were treated for 24 h with either 1.0 μM (Panel (A)) or 5.0 μM (Panel (B)) MnPs and 1.0 mM ascorbate. All conditions were, as described in Figure 2. Viability was determined by the MTT assay. Data are presented as mean ± S.D.

Our previous studies have shown that MnPs catalyze ascorbate oxidation one-electronically (with a deprotonation step) into ascorbyl radical. Upon one-electron reoxidation of MnPs with oxygen, superoxide is produced which dismutes to H_2_O_2_ [8]. Since sensitivity to ascorbate alone was cell line-dependent, one would expect that the toxicity of MnP/ascorbate would be also cell line-dependent. Data displayed in Figure 3 demonstrate that pII cells were more sensitive to the combination of MnTE-2-PyP and ascorbate than MDA-MB-231 cells, but no significant difference between the two cell lines was observed when other metalloporphyrins were tested. The results presented in Figure 3 also show that at a fixed ascorbate concentration, toxicity was dependent on the concentration and the properties of the MnP. In the presence of 1.0 mM ascorbate, 5.0 μM MnTE-2-PyP killed almost 100% of pII cells and ∼50% of the MDA-MB-231 cells (Figure 3(B)). With respect to pII cell line sensitivity, the tested MnPs (5.0 μM + 1.0 mM ascorbate) can be arranged in the following order of decreasing efficacy: MnTE-2-PyP > MnTPhE-2-PyP > MnTnOct-2-PyP = MnTnHexOE-2-PyP > MnTE-2-PyPhP. Similar results were obtained for the MDA-MB-231 cell line.

**Figure 4.**
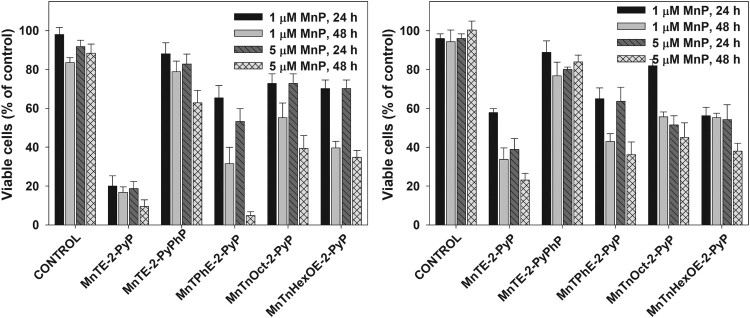
Effect of MnP/ascorbate treatment on viable cell count. Note: Cells were treated with 1.0 mM ascorbate and 1 or 5 µM MnP. Viable cell number was determined 24 and 48 h after treatment using the SRB assay. Panel (A), pII cells; Panel (B), MDA-MB-231 cells. Mean ± S.D. is presented.

As mentioned before, the MTT assay reflects metabolic activity [34]. Yet, the suppression of metabolism does not necessarily indicate loss of viability. Another way to assess the extent of cell damage is by determining the number of attached, proliferating cells. Figure 4(A) shows that when combined with ascorbate, MnTE-2-PyP dramatically decreased the number of viable pII cells. The effect was much more pronounced 48 h after the treatment. A similar result was observed when pII cells were treated with 5 µM MnTPhE-2-PyP and ascorbate for 48 h. If taking only 48 h of incubation into consideration, the tested MnPs can be arranged in the following order of decreasing cytotoxicity toward pII cells: MnTE-2-PyP ≥ MnTPhE-2-PyP > MnTnHexOE-2-PyP ≥ MnTnOct-2-PyP > MnTE-2-PyPhP.

A comparison between panels (A) and (B) (Figure 4) reveals that MDA-MB-231 cells were more resistant to MnP/ascorbate treatment. If compared at 48 h after the treatment, the tested MnPs can be arranged in the following order of decreasing cytotoxicity toward MDA-MB-231 cells: MnTE-2-PyP > MnTPhE-2-PyP ≥ MnTnHexOE-2-PyP = MnTnOct-2-PyP > MnTE-2-PyPhP. The results demonstrate that not only the two cell lines display different sensitivity toward the combination of MnP with ascorbate, but they respond differently to the individual MnPs. Under all tested conditions, MnTE-2-PyPhP displayed the lowest cytotoxicity as a result of its lowest ability to catalyze ascorbate oxidation and to generate hydrogen peroxide.

Besides catalyzing ascorbate oxidation with the production of H_2_O_2_, MnPs decompose H_2_O_2_ (Table 1) [23], and could thus eventually protect the cells. Therefore, the cytotoxic effect of MnPs would depend not only on their ability to catalyze ascorbate oxidation thus H_2_O_2_ production, but also on their peroxidase/catalase-like activity [23,39].

**Figure 5.**
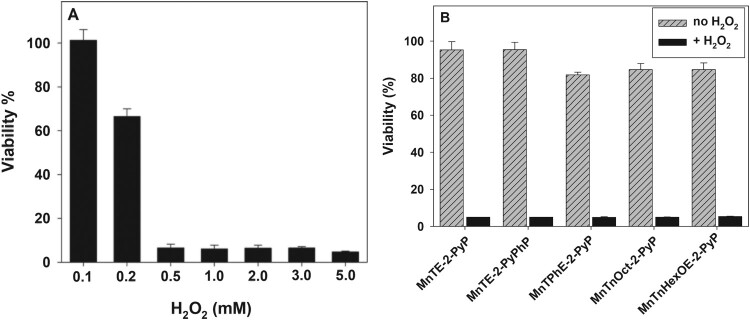
Effect of MnPs on the cytotoxic action of hydrogen peroxide. Note: Cytotoxicity of hydrogen peroxide on pII cells and assessment of possible peroxidase activity of MnP. PII cells were treated for 24 h with varying concentrations of H_2_O_2_ (Panel (A)) or a combination of 1.0 mM H_2_O_2_ and 5 μM MnP (Panel (B)). Viability was determined using the MTT assay. Mean ± S.D. is shown.

In order to test the effect of MnPs on H_2_O_2_ decomposition, cell cultures were treated with H_2_O_2_ with or without the presence of MnP. Incubation of pII cells, with varying concentrations of H_2_O_2_ alone, revealed that 0.2 mM H_2_O_2_ caused ∼30% loss of viability. At a concentration of 0.5 mM or higher, practically 100% of the cells were killed (Figure 5(A)). None of the tested MnPs; however, suppressed H_2_O_2_ cytotoxicity under our experimental conditions (Figure 5(B)).

**Figure 6.**
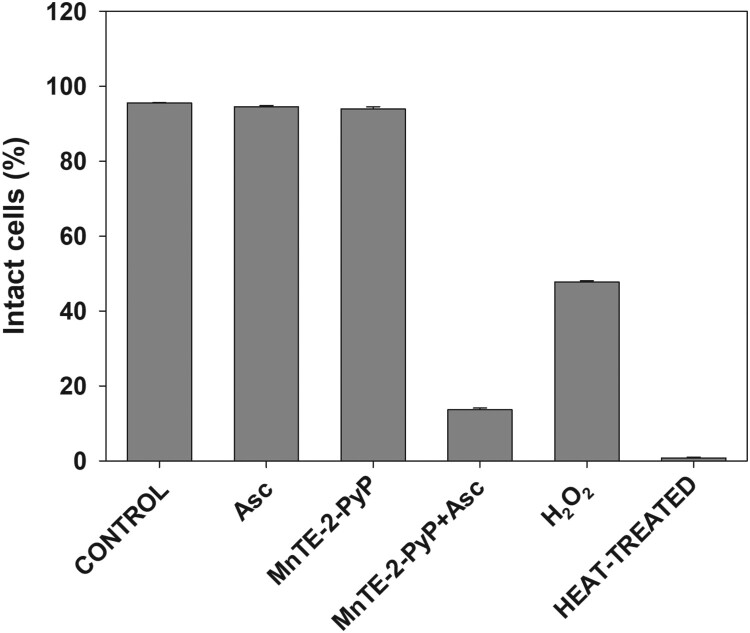
Effect of MnP/ascorbate treatment on plasma membrane integrity. Note: Loss of membrane integrity of pII cells after MnP + ascorbate treatment. Cells were incubated for 24 h with the listed compounds. Plasma membrane permeability was determined by propidium iodide staining and flow cytometer. Data are presented as mean ± S.D.

Results presented so far suggest that reactive species generated by MnPs-catalyzed ascorbate oxidation damage structures that are essential for cell survival. Among such structures is the plasma membrane. It has been reported that when combined with ascorbate, MnPs damage cells from the outside [4,6]; therefore, plasma membrane would be the first critical cellular structure attacked by reactive species. Data presented in Figure 6 show that upon treatment with MnTE-2-PyP + ascorbate, which displayed the highest cytotoxicity, plasma membrane integrity was almost completely lost.

**Figure 7.**
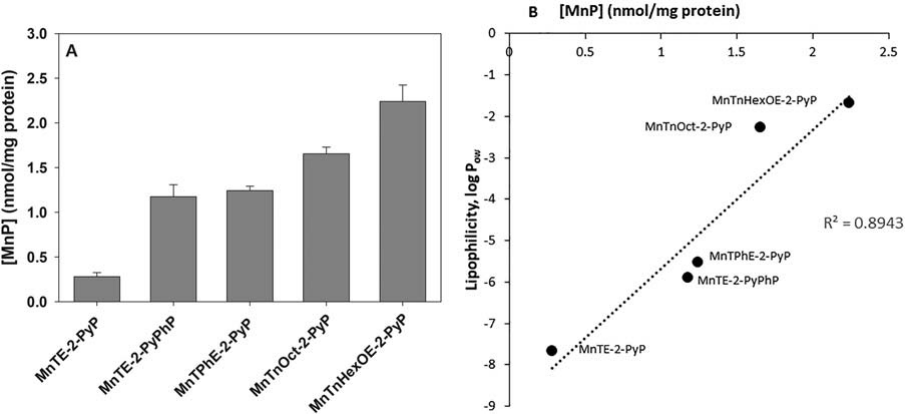
Uptake of MnPs by pII cells. Note: PII cells were incubated for 24 h with 5 µM MnPs and after thorough washing were solubilized by treatment with 2% SDS. Intracellular MnP concentration was determined spectrophotometrically, as described in the Materials and Methods section. (A) Total MnP content in nmol per mg protein; (B) Relationship between lipophilicity and cellular MnP uptake. Data are presented as mean ± S.D.

Cancerous tissues selectively accumulate porphyrins, a property that was initially used to visualize tumors, and was later applied in selective tumor eradication by photodynamic therapy [16]. By analogy, selective anticancer treatment, by a combination of a MnP and natural reductants, could be achieved if MnPs taken up by cancer cells redox-cycle and generate intracellularly toxic levels of ROS. Figure 7 demonstrates that after 24 h of incubation with MnPs, pII cells accumulate between ∼0.3 and ∼2.3 nmoles of MnPs per mg protein, depending on the lipophilicity of the compound. Uptake was the highest for the most lipophilic MnTnHexOE-2-PyP and the lowest for the most hydrophilic MnTE-2-PyP. It can be expected that if MnPs, which accumulate to the highest levels in cancer cells, are able to redox-cycle intracellularly, they could generate ROS at a rate sufficient to inflict irreversible cell damage and consequently, cell death. In order to test such a hypothesis, cell cultures were preincubated with MnPs for 24 h, the cells were washed to remove the unbound metalloporphyrins, and ascorbate was then added.

**Figure 8.**
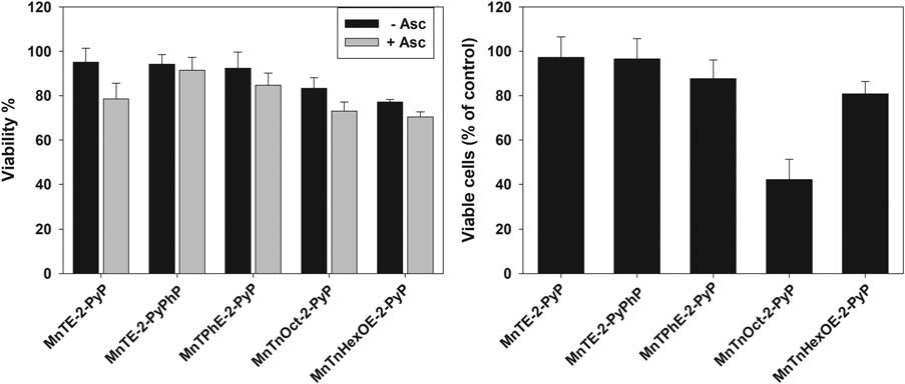
Cytotoxic effect of MnPs taken up by the cells. Note: Cells were incubated for 24 h with 5 µM MnPs and wells were washed with PBS to remove unbound compounds. Fresh medium or, where indicated, medium containing 1 mM ascorbate was added to each well. Panel (A): After 24 h of incubation cell viability was determined by the MTT assay. Panel (B): Number of viable cells was determined by the SRB assay 48 h after the removal of the unbound compounds. Mean ± S.D. is shown.

Data presented in Figure 8(A) show that when cells were preincubated with 5 μM MnPs for 24 h prior to the addition of ascorbate, small inhibition of MTT reduction (∼ 20%) was observed by MnTnOct-2-PyP and MnTnHexOE-2-PyP, but it was ascorbate independent. At a concentration of 10 μM, MnTnOct-2-PyP suppressed MTT reduction by 55 ± 5%, and MnTnHexOE-2-PyP, by 47 ± 4% (not shown). Addition of ascorbate again did not have a significant effect. These two compounds are the most lipophilic and Figure 7 shows that MnTnOct-2-PyP and MnTnHexOE-2-PyP accumulate to the highest levels in pII cells. The hydrophilic ethyl analog was taken up much less, which explains its low cytotoxicity under these experimental conditions and confirms that it exerts its effect extracellularly [4,6].

When cells were treated with 5 μM MnPs for 24 h and viable cells were counted 48 h after the treatment, the biggest decrease of viable cells (∼60%) was found in the samples exposed to MnTnOct-2-PyP (Figure 8(B)). Irrespective of its highest accumulation in the cells, MnTnHexOE-2-PyP was much less efficient (∼20% decrease of viable cell number). Externally added ascorbate had no effect under these experimental conditions (not shown).

## Discussion

When cancer cell viability was explored by the metabolically dependent MTT assay the most cytotoxic compound in combination with ascorbate was MnTE-2-PyP, followed by MnTPhE-2-PyP. As previously reported, MnTE-2-PyP is closest to the optimal reduction potential for cycling with ascorbate [8] and is most active in catalyzing ascorbate oxidation (Table 1). MnTPhE-2-PyP comes closest to MnTE-2-PyP with respect to the redox potential and rate of ascorbate oxidation. These two compounds, when combined with ascorbate, also caused the strongest suppression of cell proliferation.

**Figure 9.**
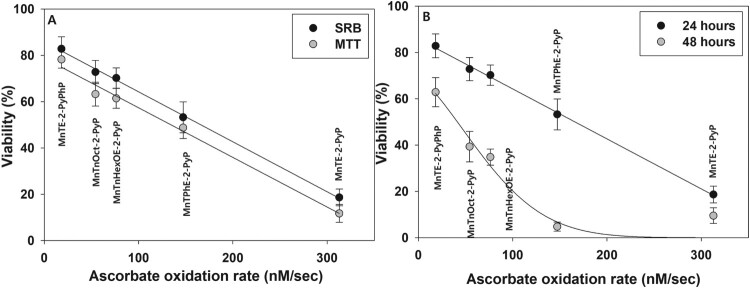
Correlation between ascorbate oxidation rate and MnPs cytotoxicity. Note: Panel (A): pII cells were treated for 24 h with 5.0 μM MnPs + 1.0 mM ascorbate and viability was determined by the MTT or SRB assay. Panel (B): pII cells were treated as in Panel (A), and viable cell number was determined by the SRB assay 24 or 48 h after the treatment.

In the presence of externally added ascorbate, cytotoxicity of the MnP tested correlated well with their ability to catalyze ascorbate oxidation (Figure 9). This proves that in the presence of a reductant, MnPs exert cytotoxicity by redox-cycling and generating cell-damaging ROS.

As mentioned before, it was established that in cell culture experiments, MnP/ascorbate damages cells predominantly by generating ROS in the medium, outside of the cells [4]. Such mechanism of action lessens the main advantage of metalloporphyrins, their high selective uptake and accumulation in tumors, which is the basis of targeted cancer therapy. In an attempt to increase the uptake of MnPs by the cancer cells, metalloporphyrins were modified by attaching substituents of varying lipophilicity to the *meso* pyridyl nitrogens without altering the tetrapyrrolic core of the compound. Such modifications affected MnPs’ lipophilicity, size, three-dimensional shape, and reduction potential (Table 1). Due to the steric hindrance, the long-chain substituted MnPs display a low rate of ascorbate oxidation [8]. Shift of the reduction potential away from its optimal value and steric hindrance decreased the anticancer activities of the compounds when acting in combination with ascorbate. Due to its negative reduction potential (*E*_½_ = −65 mV), MnTE-2-PyPhP was the least cytotoxic. At the same time, addition of lipophilic substituents increased the uptake and accumulation of MnPs in the cells, but this did not increase cell killing by externally added ascorbate.

**Figure 10.**
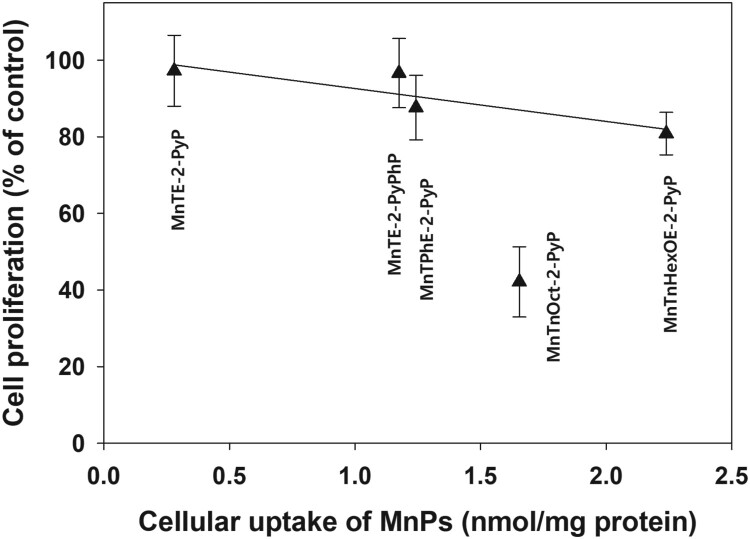
Correlation between cellular uptake and cytotoxicity of MnPs.

Increasing lipophilicity, however, resulted in increased cytotoxicity in the absence of added ascorbate. When viable cell number was determined 48 h after the removal of the compounds, ∼60% of cell loss was observed in samples treated with MnTnOct-2-PyP (Figure 10). Figure 10 demonstrates that, other than with MnTnOct-2-PyP, cellular uptake of MnPs correlates well with their ascorbate-independent cytotoxicity. If MnTnOct-2-PyP is excluded, cytotoxicity depends linearly on the cellular uptake of the compounds.

**Scheme 1.**
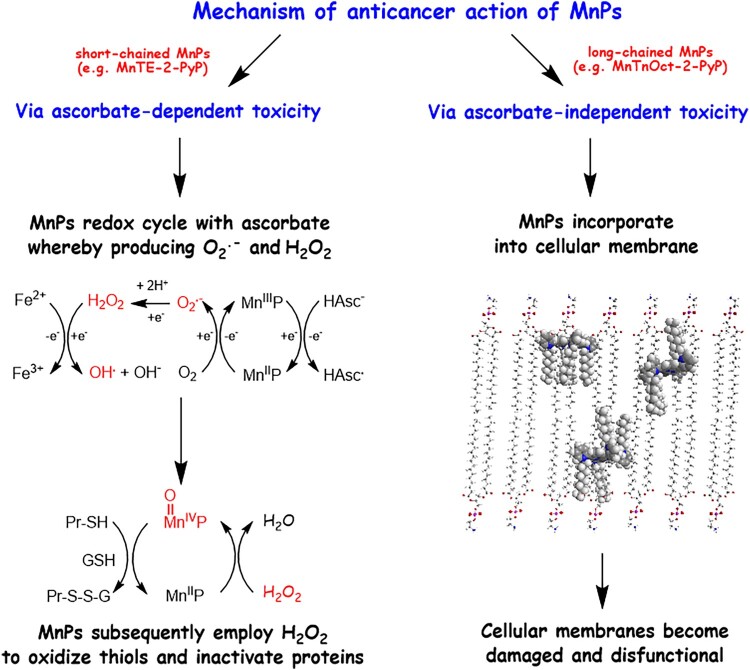
The anticancer effects of Mn porphyrins. Note: Mn porphyrins exhibit anticancer effects via at least two pathways described here and reported elsewhere in detail. MnPs that bear short chains and have appropriate *E*_1/2_, such as MnTE-2-PyP, are the most efficient catalysts of ascorbate oxidation. Because such compounds are hydrophilic, their uptake and incorporation in cells is limited. Such MnPs exert cytotoxicity primarily by redox-cycling with natural reductants, such as ascorbate (under physiological conditions ascorbate is present in its monodeprotonated form HAsc^−^), thus generating H_2_O_2_ and other ROS. Hydrogen peroxide can be decomposed to hydroxyl radical, which can attack and damage practically all biomolecules. In addition, H_2_O_2_ can oxidize Mn(II)P to a strong oxidizer, O = Mn(IV)P which also can oxidize biomolecules, including thiols [8,9,43]. MnPs that bare long aliphatic chains are lipophilic and accumulate in the lipid bilayer of cell membranes. Such MnPs are poor catalysts of ascorbate oxidation, but depending on their three-dimensional shape, flexibility, bulkiness, and strength of hydrophobic interactions, can compromise membrane integrity and essential functions, ultimately causing cell death.

Irrespective of its lower lipophilicity and lower uptake by the cancer cells than MnTnHexOE-2-PyP, MnTnOct-2-PyP was much more effective in suppressing cell proliferation. This finding suggests that in addition to uptake, localization of a MnP to critical cellular structures is essential for cell damage and death. Due to hydrophobic aliphatic chains of 8 carbon atoms attached to the positively charged pyridyl nitrogen, MnTnOct-2-PyP may easily intercalate and distort the membrane lipid bilayer (Scheme 1). Our previous investigations demonstrated that subcellular distribution of analogous ZnPs depended on the length and position of the aliphatic chains attached to the pyridyl nitrogens [19]. Increasing the length of the aliphatic chains form four (butyl) to six (hexyl) carbons increased accumulation in mitochondria and plasma membrane [19,20]. Distribution to plasma membrane was further increased by moving the hexyl chain form *ortho* to *para* position, which increased lipophilicity by three orders of magnitude [19]. MnTnOct-2-PyP is about tenfold more lipophilic than its hexyl analog [40], and therefore would be more soluble in the membrane lipid bilayer. It has been also established that the structure of metalloporphyrin molecule controls the depth of penetration within the lipid bilayer of membranes and the strength of hydrophobic interactions [41]. Insertion of MnTnOct-2-PyP in membranes would distort the lipid bilayer preventing the tight lipid packing and creating gaps between neighboring phospholipid molecules (Scheme 1). This would compromise the membrane integrity and barrier functions, leading to cell death.

Introduction of oxygen atoms in the MnTnHexOE-2-PyP molecule disrupts hydrophobic interactions and consequently, suppresses dispersion of the compound in biomembranes. The effect of introducing a polar group in the aliphatic chains, attached at the periphery of the porphyrin ring, is well illustrated by *in vivo* study with Mn(III) *meso*-tetrakis(*N*-n-hexylpyridinium-2-yl)porphyrin (MnTnHex-2-PyP), and its analog Mn(III) *meso*-tetrakis(*N*-n-butoxyethylpyridinium-2-yl)porphyrin (MnTnBuOE-2-PyP) [42]. Irrespective of the fact that these two MnPs have a similar structure and the same number of atoms of the chains attached to the meso pyridyl nitrogen, introduction of oxygen atom in those chains largely affected brain tissue distribution and systemic toxicity of the two compounds.

In conclusion, peripheral modifications of MnPs, which increase the lipophilicity and the cellular uptake, shift reduction potential away from the optimal value which would have provided fast redox cycling with natural reductants, and production of cytotoxic reactive species. Irrespective of this shortcoming, MnPs that are efficiently taken up by cancer cells, and can interact with and modify critical cellular targets, can exert cytotoxic action. If such compounds are preferentially taken up by cancerous tissues, they could be better drugs for selective anticancer treatment.
